# Quality of Life Among Caregivers of Patients Undergoing Hemodialysis Versus Peritoneal Dialysis in Saudi Arabia: A Cross-Sectional Study

**DOI:** 10.7759/cureus.77834

**Published:** 2025-01-22

**Authors:** Hatem A Alnasser, Yasser A BinMuneif, Saud F Alrsheed, Sultan A Alqahtani, Faisal E Alhaisoni, Homoud A Algadheb, Naif M Alateeq

**Affiliations:** 1 Department of Medicine, King Saud University, Riyadh, SAU; 2 College of Medicine, King Saud University, Riyadh, SAU; 3 College of Medicine, King Saud University, Riyadh , SAU

**Keywords:** caregiver quality of life, chronic kidney disease, cross-sectional study, hemodialysis, peritoneal dialysis, saudi arabia, zarit burden questionnaire

## Abstract

Background and objectives

Chronic kidney disease (CKD) is a global health issue affecting millions of people worldwide. The role of caregivers in the management of CKD patients on dialysis cannot be overstated. This study investigates the quality of life among caregivers of patients undergoing hemodialysis (HD) and peritoneal dialysis (PD) for CKD at King Khalid University Hospital, Riyadh, Saudi Arabia. It aims to assess the quality of life and prevalence of depression among caregivers in each dialysis modality. By recognizing significant differences in caregiving experiences between HD and PD, this research seeks to enhance understanding of caregiver burdens and overall quality of life.

Materials and methods

A cross-sectional study was conducted at King Khalid University Hospital and included 80 Saudi adult caregivers of CKD patients on HD or PD receiving treatment at the dialysis center in February 2023. Caregivers were recruited through phone surveys. Participants were required to be over 15 years old, free from psychiatric illnesses, co-residing with the patient, and providing care for at least one month.

Results

Among the 80 caregivers, 49 (61.25%) were female. A statistically significant gender distribution was observed between groups, with a higher proportion of females among PD caregivers (31 (73.81%)) compared to HD caregivers (18 (47.37%); p=0.015). The mean age of caregivers was 41.41 years (SD=12.82). Regarding education, 55 (68.75%) caregivers had a university degree or higher, with a significantly greater proportion among PD caregivers (34 (80.95%)) than HD caregivers (21 (55.26%); p=0.047).

Depression assessments showed that 54 (67.5%) caregivers experienced minimal depression, with no significant difference between groups. However, caregivers of PD patients reported lower levels of burden, with 25 (59.5%) experiencing no to mild burden compared to 14 (36.8%) of HD caregivers, showing a trend toward significance (p=0.053). Additionally, a larger proportion of PD caregivers (30 (71.43%)) were involved in long-term care (>24 months) compared to HD caregivers (17 (44.74%)), suggesting a potentially more sustainable caregiving role (p=0.057).

Conclusion

This study indicates that the quality of life for PD caregivers is generally better than that for HD caregivers, primarily due to the lower burden and higher educational level among PD caregivers. The in-center nature and frequent treatment schedule associated with HD may lead to higher levels of burden. Addressing the specific needs of caregivers based on dialysis modality can improve quality of life and enhance CKD management strategies, ultimately benefiting both patients and caregivers.

## Introduction

Chronic kidney disease (CKD) is a major public health concern globally, with an estimated worldwide prevalence of 13.4% (11.7-15.1%) [1]. It is irreversible and progressive, accompanied by an increased risk of cardiovascular diseases and many comorbidities. Patients often remain asymptomatic for most of the disease course, experiencing complications associated with kidney dysfunction only in the later stages [2]. CKD is defined by kidney damage or an estimated glomerular filtration rate (eGFR) of less than 60 mL/min/1.73 m² lasting for at least three months, regardless of the underlying cause. This gradual decline in kidney function frequently results in the necessity for renal replacement therapy, including dialysis or transplantation [3]. Factors such as a low number of nephrons at birth, nephron loss associated with aging, and acute or chronic kidney injuries due to toxic exposures or conditions like obesity and type 2 diabetes mellitus contribute to the development and progression of CKD [4].

End-stage renal disease (ESRD) necessitates dialysis as the final stage of treatment, which can be performed via hemodialysis (HD) or peritoneal dialysis (PD). Hemodialysis is the most widely used treatment globally, particularly in developed countries. However, peritoneal dialysis is increasingly recognized as a significant alternative in low- and middle-income regions, as it has proven to be the most cost-effective dialysis option. In fact, two out of three patients undergoing PD reside in developing countries [5,6]. Both treatment modalities significantly improve patient survival and well-being [7,8], but they also impose substantial physical, psychological, and social burdens on patients and their families. While much attention has been given to the clinical outcomes and quality of life (QoL) of dialysis patients, there is growing recognition of the significant impact on caregivers, who provide crucial emotional and practical support.

The quality of life in patients undergoing dialysis has been reported to be poorer than that of the general population [9]. This can be attributed to the nature of the disease and the high maintenance required for treatment. Caregivers of ESRD patients play a crucial role in patient management. A strong social support system has been shown to reduce hospitalizations of ESRD patients by 15% while improving treatment adherence and overall quality of life [10,11].

Despite the growing awareness of the challenges faced by caregivers, comprehensive studies directly comparing the quality of life of those caring for hemodialysis and peritoneal dialysis patients are lacking. Existing literature has not clearly delineated the factors contributing to caregiver burden or how these factors differ across dialysis modalities. Moreover, caregivers' needs and experiences are often overlooked in clinical settings, despite their integral role in supporting dialysis patients [10,11].

Understanding the impact of dialysis treatment on caregivers' QoL is essential, as caregiver well-being is closely linked to the health and well-being of the patient. One study reported that 52% of caregivers for hemodialysis patients have moderate to low levels of quality of life [12], with 67.5% reporting no recreational activities in their daily lives [12]. High levels of caregiver stress and burden can negatively affect the patient's treatment adherence and overall quality of life [11]. By identifying the unique challenges and stressors faced by caregivers of hemodialysis and peritoneal dialysis patients, healthcare providers can better tailor support systems and interventions to alleviate caregiver burden and improve the overall care experience for both patients and their families.

## Materials and methods

This cross-sectional study was conducted at King Khalid University Hospital (KKUH), Riyadh, Saudi Arabia, targeting adult male and female caregivers of dialysis patients. The study aimed to explore the burden among caregivers of patients on HD and PD. An estimated sample size of 80 was calculated based on a 5% margin of error and a 95% confidence level. Participants were identified using contact information provided by the KKUH dialysis unit, which included the caregivers' phone numbers. Caregivers were contacted via phone, during which the study was explained to them in detail, and informed consent was obtained. Once consent was granted, caregivers were sent a link to an electronic survey via their mobile phones, allowing them to complete the survey independently at their convenience. This method ensured both convenience and flexibility while respecting participant privacy and their right to withdraw from the study at any stage. Inclusion criteria specified that participants must be over 15 years old, have been residing with patients on dialysis for at least one month, and have been providing care for at least one month. Participants were also required to be free from any psychiatric illness. Any participant who did not meet these criteria was excluded.

The survey collected data on demographic factors, dialysis duration, and other variables necessary for the analysis. Additionally, the Arabic Zarit Burden Interview-short version (ZBI A) was employed to measure caregiver burden. A five-point Likert scale was used to rate 12 items, ranging from 0, representing "almost never," to 4, representing "almost always." Scores ranged from 0 to 48, with a higher score indicating greater burden [13]. A score of 17 or more was categorized as a high burden level [14,15]. The ZBI A demonstrated good validity and reliability (Cronbach’s alpha = 0.77) [13].

Patient health questionnaire-9 (PHQ-9)

Depression levels among caregivers were measured using the PHQ-9. This is a widely used self-administered instrument for assessing depressive symptoms across different populations [16]. It consists of nine items, each rated on a four-point Likert scale ranging from 0 (not at all) to 3 (nearly every day), resulting in a total score ranging from 0 to 27. A score of 10 or higher indicates the presence of depressive symptoms. The study utilized the Arabic version of the PHQ-9 to ensure accuracy for Saudi caregivers. This version has been validated as a reliable tool for screening depression in the Saudi population, with good internal consistency and a Cronbach’s alpha of 0.85 [17].

Ethical considerations

Approval was obtained from the King Saud University Institutional Review Board (KSU-IRB) (Approval No. E-24-8573, Date: February 12, 2024). All participants' information was handled with strict confidentiality, with patient identifiers removed during data analysis and publication to safeguard privacy.

## Results

The study included 80 caregivers, with 31 (38.75%) males and 49 (61.25%) females. A statistically significant difference was observed in gender distribution between caregivers of HD and PD patients (p=0.015), with a higher proportion of males in the HD group (52.63%) and females in the PD group (73.81%).

Educational levels varied among caregivers: 6 (7.5%) had less than a high school education, 19 (23.75%) were high school graduates, and 55 (68.75%) had a university degree or higher. A statistically significant difference (p=0.047) was found between the groups, with caregivers of PD patients having a higher proportion of university-educated individuals (80.95%) compared to those in the HD group (55.26%).

Regarding the relationship to the patient, 44 (55%) of caregivers were daughters or sons, 16 (20%) were spouses or partners, and 17 (21.25%) were parents or siblings, with no significant group differences (p=0.860). Marital status indicated that 50 (62.5%) caregivers were married, with no significant differences between HD and PD groups (p=0.729). Employment status showed that 46 (57.5%) were employed, while 34 (42.5%) were unemployed, with no significant differences between groups (p=0.700). Monthly income revealed that 35 (43.75%) caregivers earned less than 5000 SR, with smaller proportions in higher income brackets and no significant differences between HD and PD groups (p=0.849). For dialysis duration, 47 (58.75%) caregivers cared for patients on dialysis for more than 24 months, showing no significant group differences (p=0.057) (Table 1).

**Table 1:** Socio-demographic characteristics of caregivers (N=80) This table summarizes the socio-demographic characteristics of caregivers, including gender, age, education level, relationship to the patient, marital status, employment, income, and duration of caregiving. It compares caregivers of hemodialysis (HD) and peritoneal dialysis (PD) patients, highlighting significant differences in gender and education level. * Significant p-value.

Characteristics	Category	All (n=80)		Hemodialysis (n=38, 47.5%)		Peritoneal dialysis (n=42, 52.5%)		p- value
		Number	%	Number	%	Number	%	
Gender	Male	31	38.75	20	52.63	11	26.19	0.015*
	Female	49	61.25	18	47.37	31	73.81	
Age (mean, SD)		41.41	12.82	42.32	13.27	40.60	12.50	0.55
Educational level	Less than high school	6	7.50	4	10.53	2	4.76	0.047*
	High school	19	23.75	13	34.21	6	14.29	
	University and above	55	68.75	21	55.26	34	80.95	
Relationship to patient	Spouse/partner	16	20.00	8	21.05	8	19.05	0.860
	Daughter/son	44	55.00	21	55.26	23	54.76	
	Parent/sibling	17	21.25	7	18.42	10	23.81	
	Others (driver, nurse)	3	3.75	2	5.26	1	2.38	
Marital status	Married	50	62.50	23	60.53	27	64.29	0.729
	Unmarried	30	37.50	15	39.47	15	35.71	
Job	Employed	46	57.50	21	55.26	25	59.52	0.700
	Unemployed	34	42.50	17	44.74	17	40.48	
Monthly income	<5000 SR	35	43.75	18	47.37	17	40.48	0.849
	5000-10,000 SR	22	27.50	11	28.95	11	26.19	
	10,000-15,000 SR	7	8.75	3	7.89	4	9.52	
	15,000-20,000 SR	9	11.25	4	10.53	5	11.90	
	>20,000 SR	7	8.75	2	5.26	5	11.90	
Duration of dialysis in months	<3	2	2.50	1	2.63	1	2.38	0.057
	3-6	8	10.00	7	18.42	1	2.38	
	6-12	14	17.50	9	23.68	5	11.90	
	12-24	9	11.25	4	10.53	5	11.90	
	>24	47	58.75	17	44.74	30	71.43	

**Figure 1:**
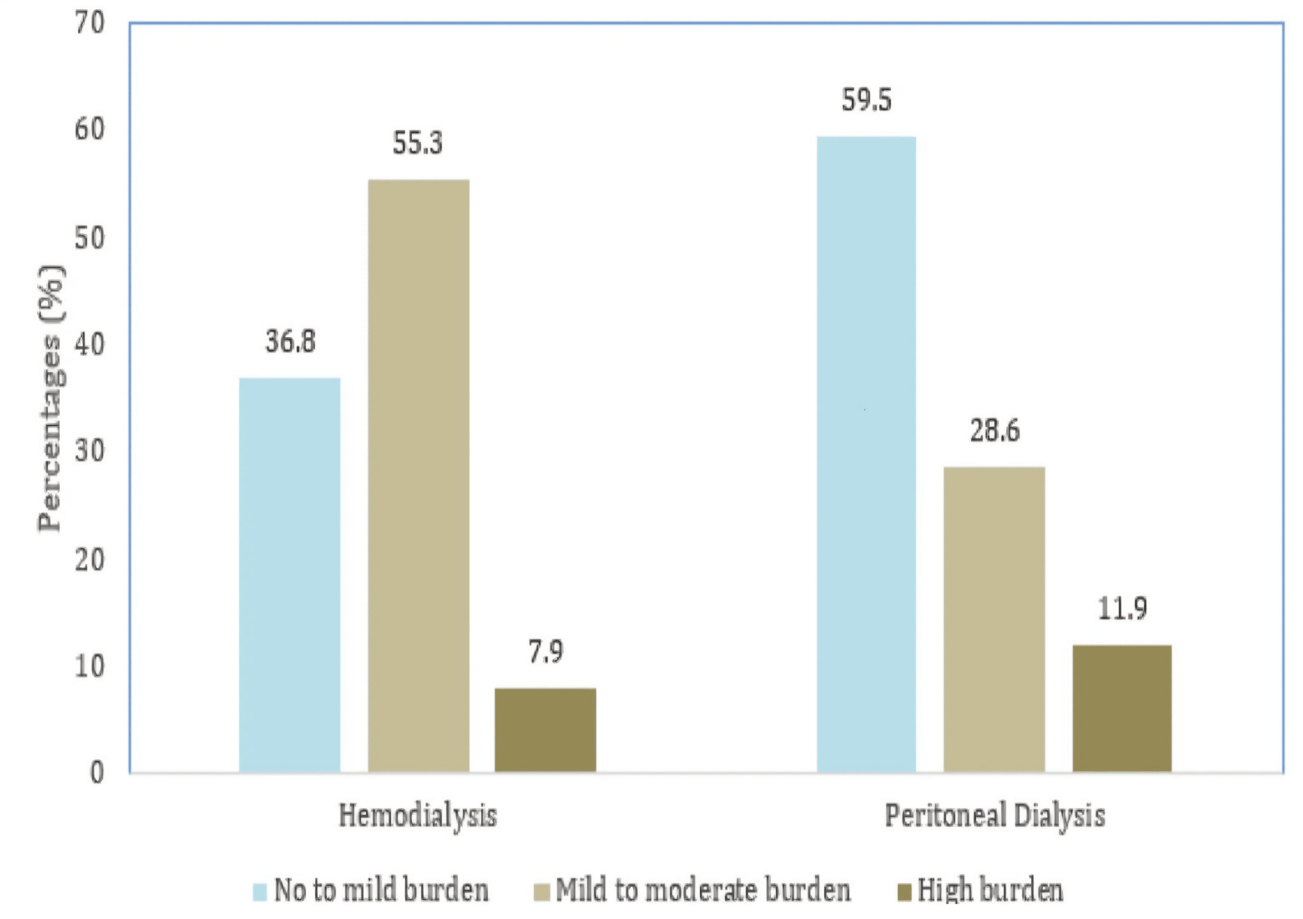
Assessment of burden in caregivers of patients undergoing hemodialysis (HD) and peritoneal dialysis (PD) using Zarit Burden Interview 12 This figure illustrates the distribution of caregiver burden levels (no to mild, mild to moderate, and high) among caregivers of HD and PD patients. HD caregivers report a higher mild to moderate burden compared to PD caregivers, while no to mild burden is more common in PD caregivers.

The Zarit Burden Interview (ZBI-12) tool indicated that 39 (48.8%) caregivers reported no to mild burden, 33 (41.3%) reported mild to moderate burden, and 8 (10%) experienced a high burden. Among HD caregivers, 21 (55.3%) reported mild to moderate burden compared to 12 (28.6%) of PD caregivers, with a trend toward significance (p=0.053) (Table 2 and Figure 1).

**Table 2:** Assessment of burden in caregivers of patients undergoing hemodialysis and peritoneal dialysis using ZBI-12 This table shows the levels of caregiver burden (no to mild, mild to moderate, and high) based on the Zarit Burden Interview (ZBI-12). It compares the burden in caregivers of hemodialysis and peritoneal dialysis patients, indicating a higher proportion of mild to moderate burden among HD caregivers.

ZBI-12	All (n=80)		Hemodialysis (n=38,47.5%)		Peritoneal dialysis (n=42,52.5%)		p-value
	Number	%	Number	%	Number	%	
No to mild burden	39	48.8	14	36.8	25	59.5	0.053
Mild to moderate burden	33	41.3	21	55.3	12	28.6	
High burden	8	10.0	3	7.9	5	11.9	

**Figure 2:**
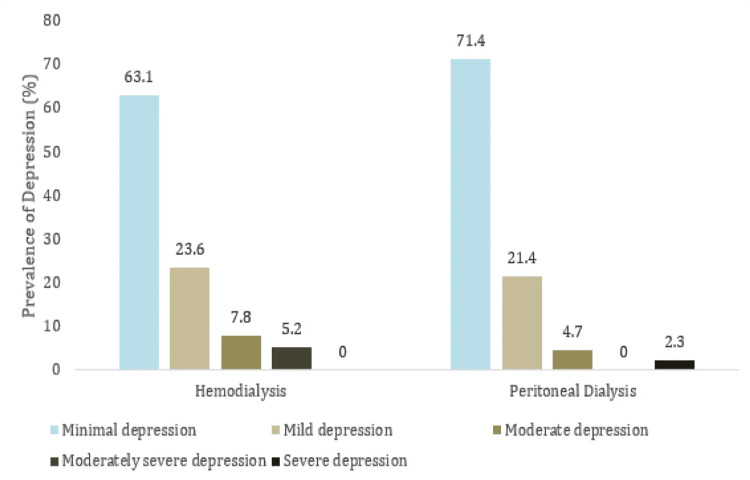
Assessment of the prevalence of depression in primary caregivers of patients undergoing regular dialysis using Patient Health Questionnaire-9 This figure visualizes the severity of depression (minimal to severe) among caregivers of hemodialysis (HD) or peritoneal dialysis (PD) patients. Minimal depression is more prevalent in PD caregivers, while moderate and severe depression is slightly higher among HD caregivers.

Depression levels among caregivers, assessed using the PHQ-9, showed that 54 (67.5%) had minimal depression, 18 (22.5%) had mild depression, 5 (6.25%) had moderate depression, 2 (2.5%) had moderately severe depression, and 1 (1.25%) had severe depression. No statistically significant differences were found between HD and PD caregivers (p=0.452) (Table 3 and Figure 2).

**Table 3:** Assessment of the prevalence of depression in primary caregivers of patients undergoing regular dialysis using Patient Health Questionnaire-9 This table presents the prevalence of depression severity (minimal, mild, moderate, moderately severe, and severe) among caregivers of hemodialysis (HD) and peritoneal dialysis (PD) patients, assessed using the PHQ-9. The majority of caregivers reported minimal depression, with a slightly higher prevalence of moderate to severe depression in HD caregivers.

Depression severity	All (n=80)		Hemodialysis (n=38,47.5%)		Peritoneal dialysis (n=42,52.5%)		p-value
	Number	%	Number	%	Number	%	
Minimal depression	54	67.50	24	63.16	30	71.43	0.452
Mild depression	18	22.50	9	23.68	9	21.43	
Moderate depression	5	6.25	3	7.89	2	4.76	
Moderately severe depression	2	2.50	2	5.26	0	0.00	
Severe depression	1	1.25	0	0.00	1	2.38	

Mean scores for individual ZBI-12 items reflected varying levels of caregiver burden, with notable scores for feeling the need to do more for their relative (mean = 2.76, SD = 1.40) and feeling stressed between caregiving and other responsibilities (mean = 1.61, SD = 1.43). The total burden score was 11.26 (SD = 7.37) (Table 4).

**Table 4:** Mean score for items of Zarit Burden Interview (ZBI-12) questionnaire This table provides the mean scores for each item in the ZBI-12 questionnaire, highlighting the specific areas of burden experienced by caregivers. The highest scores relate to feelings of inadequacy in caregiving and the need to do more for the patient.

Zarit burden items	N	Minimum	Maximum	Mean	SD
That because of the time you spend with your relative that you don’t have enough time for yourself?	80	0	4	1.55	1.37
Stressed between caring for your relative and trying to meet other responsibilities (work/family)?	80	0	4	1.61	1.43
Angry when you are around your relative?	80	0	3	0.23	0.64
That your relative currently affects your relationship with family members or friends in a negative way?	80	0	3	0.16	0.56
Strained when you are around your relative?	80	0	4	0.38	0.83
That your health has suffered because of your involvement with your relative?	80	0	4	0.56	1.03
That you don’t have as much privacy as you would like because of your relative?	80	0	2	0.31	0.67
That your social life has suffered because you are caring for your relative?	80	0	4	0.48	0.89
That you have lost control of your life since your relative’s illness?	80	0	4	0.64	1.09
Uncertain about what to do about your relative?	80	0	4	0.50	0.94
You should be doing more for your relative?	80	0	4	2.76	1.40
You could do a better job in caring for your relative?	80	0	4	2.09	1.56
Total score of Zarit burden	80	0	38	11.26	7.37

Responses indicated that 54 (67.5%) caregivers experienced little interest or pleasure in activities "not at all," while 21 (26.25%) reported feeling this way "several days." The majority, 79 (98.75%), did not report suicidal thoughts. Other symptoms, such as trouble sleeping and feeling tired, varied in frequency among respondents (Table 5).

**Table 5:** Responses of the caregivers about the Patient Health Questionnaire-9 (PHQ-9) questionnaire This table presents caregiver responses to the PHQ-9 questionnaire, detailing the frequency of symptoms such as loss of interest, feelings of depression, trouble sleeping, and fatigue. The data are categorized by frequency (not at all, several days, more than half the days, and nearly every day).

PHQ-9 items	Not at all		Several days		More than half the days		Nearly every day	
Questions	Number	%	Number	%	Number	%	Number	%
Little interest or pleasure in doing things	54	67.50	21	26.25	4	5.00	1	1.25
Feeling down, depressed, or hopeless	48	60.00	28	35.00	2	2.50	2	2.50
Trouble falling or staying asleep, or sleeping too much	37	46.25	34	42.50	4	5.00	5	6.25
Feeling tired or having little energy	34	42.50	36	45.00	7	8.75	3	3.75
Poor appetite or overeating	41	51.25	27	33.75	7	8.75	5	6.25
Feeling bad about yourself—or that you are a failure or have let yourself or your family down	63	78.75	13	16.25	2	2.50	2	2.50
Trouble concentrating on things, such as reading the newspaper or watching television	56	70.00	18	22.50	3	3.75	3	3.75
Moving or speaking so slowly that other people could have noticed? Or the opposite—being so fidgety or restless that you have been moving around a lot more than usual	60	75.00	16	20.00	2	2.50	2	2.50
Thoughts that you would be better off dead or of hurting yourself in some way	79	98.75	1	1.25	0	0.00	0	0.00

## Discussion

The current study evaluates the level of caregiver burden experienced by individuals caring for patients undergoing either PD or HD. Our findings indicate that most caregivers reported experiencing no burden to mild burden or moderate levels of burden in both the PD and HD groups, consistent with previous findings from Alaryni et al. [18]. This outcome reflects that caregiving can be a demanding responsibility, although it does not necessarily translate to high levels of stress for all individuals. Similar to Alaryni et al. [18], our study found that a significant proportion of caregivers in both groups did experience burdens, with a somewhat greater proportion of caregivers for HD patients reporting a higher burden level. This suggests that the HD treatment regimen may add additional strains on caregivers.

According to Alaryni et al. [18], caregivers of HD patients encounter a substantial burden compared to those caring for PD patients, which aligns with our findings, although no statistically significant differences were observed between the groups in our study. This finding contrasts with other studies that reported a clear distinction in caregiver burden based on the type of dialysis treatment. For instance, Shimoyama et al. found a notably lower caregiver burden in individuals caring for PD patients compared to those caring for HD patients, with a statistically significant difference (p < 0.005) [19]. In contrast, Alwakeel et al. observed a higher caregiver burden in PD caregivers than in HD caregivers; however, their findings were not statistically significant [20]. These discrepancies suggest that while caregiver burden may differ between groups, it is influenced by factors such as caregiver support systems, dialysis settings, and individual circumstances.

The social and psychological effects of caregiving were also a focus of Sezer et al., who found that depression and somatization scores were notably higher in caregivers of HD patients than those caring for PD patients or a control group [21]. Our study similarly observed that HD caregivers reported moderate burden levels, which could be related to the emotional and physical demands of accompanying patients to dialysis centers multiple times a week. The need for HD caregivers to be present at treatment sessions may restrict their social and personal lives more significantly than for PD caregivers, who typically have more flexibility. This is consistent with Sezer et al.'s findings, which suggested that the in-center nature of HD treatment places additional constraints on caregivers, potentially impacting their mental health due to lost time for personal or social activities [21].

Both Alaryni et al. and Sezer et al. emphasize the strain caregiving places on personal and social lives [18,21]. Our findings align with these observations, as caregivers in our study reported disruptions in their personal time and social relationships. High ZBI-12 scores for items such as "strain around relatives" and "social life suffering" underscore the challenges faced by caregivers. These findings call for interventions tailored to address the unique needs of HD and PD caregivers, with a focus on time management, educational support, and psychological well-being.

Examining demographic variables, our study revealed no significant differences in caregiver burden based on age, gender, or marital status, aligning with findings from Alaryni et al. [18], which showed that these variables do not substantially affect caregiver burden in dialysis settings. However, other studies have presented contrasting views. For instance, Jafari et al. found a positive correlation between caregiver age and caregiving burden, suggesting that older caregivers may experience a greater burden [22]. Similarly, gender and marital status were shown to influence caregiver burden in other research, with factors such as female gender and being unmarried associated with higher burdens in specific contexts.

This contrast indicates that demographic factors may impact caregiver burden differently across cultural and individual settings, emphasizing the need for context-specific support systems.

Education level was another demographic factor evaluated in our study. The findings indicated a higher percentage of PD caregivers with university-level education, consistent with Alaryni et al. [18], who noted that education levels can influence caregivers' perspectives and experiences regarding burden. Rafati et al. suggested that higher education among caregivers could reduce the perceived burden, potentially due to better coping skills or an enhanced understanding of the treatment process [23]. Interestingly, Alwakeel et al. reported contrasting results, where a higher percentage of HD caregivers had university-level education compared to PD caregivers. This difference in educational attainment was linked to a lower caregiver burden in the HD group in their study [20], highlighting the critical role of education in shaping caregiver experiences. These findings emphasize that caregivers with higher education levels may better understand the caregiving process, manage stress more effectively, and develop stronger coping mechanisms, potentially mitigating their perceived burden.

These insights suggest that educational interventions targeting caregivers, regardless of dialysis type, could alleviate perceived burdens and improve their resilience. Tailored training and support programs aimed at enhancing caregivers' understanding of dialysis procedures and patient care could provide significant benefits, particularly for caregivers with lower education levels or limited healthcare literacy.

Sezer et al. emphasized the critical role of social support in mitigating caregiver burden, particularly for HD caregivers. Their findings demonstrated that caregivers with higher perceived social support exhibited improved resilience and better management of caregiving stress. Additionally, social support was linked to better treatment compliance, as caregivers with more resources could assist patients with tasks such as medical appointments and medication management [21]. These observations underscore the potential benefits of fostering robust social support networks for caregivers, particularly for those managing the demanding schedules and responsibilities associated with HD.

Caregiver burden is a multifaceted issue, with emotional and financial aspects often intertwined. Alaryni et al. highlighted that caregiving responsibilities could lead to financial strain due to reduced work hours, lost job opportunities, and missed career prospects [18]. While our study did not directly assess financial stress, the higher burden reported by HD caregivers may be influenced by these factors. This cumulative burden, combining emotional stress with economic hardship, underscores the need for financial assistance programs to support caregivers in maintaining their personal stability and well-being.

Caregiver resilience has been highlighted in several studies as a protective factor that can alleviate the negative effects of caregiving. Mashayekhi et al. found that more than half of the caregivers of HD patients reported moderate to severe burden levels [24]. While our study did not directly assess resilience, the burden reported by caregivers, particularly in the HD group, highlights the importance of interventions that focus on enhancing coping mechanisms and emotional support. This aligns with findings from Alaryni et al., who suggested that resilience training could help caregivers better navigate the challenges of caregiving for dialysis patients [18]. Developing resilience-focused interventions could therefore play a crucial role in improving the psychological and emotional well-being of caregivers.

This study has several limitations that should be acknowledged. The relatively small sample size of 80 participants limits the generalizability of the findings. A larger sample would provide a more comprehensive understanding of the differences in caregiver burden among caregivers of patients undergoing HD and PD. Moreover, the study was conducted at a single institution, KKUH in Riyadh, Saudi Arabia. Access to the KKUH dialysis unit may represent a privileged group with specific demographics or support systems, which could have influenced the makeup of the cohort studied. This introduces a potential confounding factor, as the findings may not fully represent caregivers in less privileged settings or different regions with varying healthcare access and support structures. Additionally, the cross-sectional design captures a snapshot of caregiver burden but does not allow for an exploration of changes over time or causal relationships. Longitudinal studies are needed to understand how caregiver experiences evolve throughout the caregiving journey.

## Conclusions

In conclusion, the findings of our study are largely consistent with existing literature, underscoring the significant burden faced by caregivers of patients undergoing dialysis, particularly those providing care for HD patients. While both PD and HD caregivers experience substantial responsibilities, the in-center nature and frequent treatment schedule associated with HD may lead to higher levels of burden. The results suggest that although there is no statistically significant difference in the overall burden between PD and HD caregivers, the type and extent of the burden vary depending on multiple factors, including demographic characteristics, social support, financial challenges, and individual resilience.

These findings emphasize the need for healthcare providers to implement supportive interventions tailored to caregivers' specific needs and treatment modalities. Moreover, future research should explore other contributing factors to caregiver burden, such as coping mechanisms and access to support resources. Given the physical and emotional demands of caregiving, it is crucial to develop comprehensive support systems to alleviate caregiver burden, ultimately improving outcomes for both caregivers and patients in the dialysis setting.

## Appendices

**Table 6:** Patient Health Questionnaire-9

Over the last 2 weeks, how often have you been bothered by any of the following problems? Over the last 2 weeks, how often have you been bothered by any of the following problems?	Not at all	Several days	More than half the days	Nearly every day
Little interest or pleasure in doing thingsLittle interest or pleasure in doing things​​​​​​ Little interest or pleasure in doing things	0	1	2	3
Feeling down, depressed, or hopeless​​​​​​​Feeling down, depressed, or hopeless	0	1	2	3
Trouble falling or staying asleep, or sleeping too much​​​​​​​Trouble falling or staying asleep, or sleeping too much	0	1	2	3
Feeling tired or having little energy​​​​​​Feeling tired or having little energy	0	1	2	3
Poor appetite or overeating​​​​​​Poor appetite or overeating	0	1	2	3
Feeling bad about yourself – or that you are a failure or have let yourself or your family down​​​​​​​Feeling bad about yourself – or that you are a failure or have let yourself or your family down	0	1	2	3
Trouble concentrating on things, such as reading the newspaper or watching television​​​​​​​Trouble concentrating on things, such as reading the newspaper or watching television	0	1	2	3
Moving or speaking so slowly that other people could have noticed. Or the opposite – being so fidgety or restless that you have been moving around a lot more than usual​​​​​​​Moving or speaking so slowly that other people could have noticed. Or the opposite – being so fidgety or restless that you have been moving around a lot more than usual	0	1	2	3
Thoughts that you would be better off dead, or of hurting yourself in some way​​​​​​​Thoughts that you would be better off dead, or of hurting yourself in some way	0	1	2	3
